# A Case of Recurrent Sputum-Positive Pulmonary Tuberculosis Presenting With Pulmonary Mycetoma

**DOI:** 10.7759/cureus.57757

**Published:** 2024-04-07

**Authors:** Adinath Gaikwad, Pankaj Wagh, Souvik Sarkar, Mansi Khare

**Affiliations:** 1 Medicine, Jawaharlal Nehru Medical College, Datta Meghe Institue of Higher Education and Research, Wardha, IND; 2 Respiratory Medicine, Jawaharlal Nehru Medical College, Datta Meghe Institue of Higher Education and Research, Wardha, IND

**Keywords:** computed tomography, itraconazole, sputum smear positive, active pulmonary tuberculosis, pulmonary mycetoma

## Abstract

Pulmonary tuberculosis is a notorious disease as it can cause severe morbidity and mortality. In this case, we discuss a 75-year-old male tuberculosis patient from a rural area with no underlying comorbidities who failed to continue anti-tubercular medication after two months. The case discusses the diagnostic modalities confirming the diagnosis, sputum culture for *Mycobacterium tuberculosis*, imaging studies, including X-ray and CT of the chest, and laboratory parameters for identifying pulmonary mycetoma. The patient is now on anti-tubercular therapy (isoniazid, rifampicin, pyrazinamide, and ethambutol combination) and the anti-fungal drug itraconazole. Though pharmacotherapy for the treatment of mycetoma in patients with tuberculosis has a minimal role, the more appropriate treatment is surgical excision via lobectomy. As the occurrence of tuberculosis and mycetoma is a rare phenomenon, it is essential to rule out aspergillosis as both have similar presenting symptoms. Diagnosis of this co-infection can be the crucial difference between morbidity and mortality.

## Introduction

The co-infection of pulmonary tuberculosis and aspergillosis is rare [1]. Pulmonary aspergillosis has various presentations, including allergic bronchopulmonary aspergillosis, mycetoma, necrotizing *Aspergillus *pneumonia, and invasive aspergillosis [2]. When a patient presents with tuberculosis, it is sometimes possible to miss an *Aspergillus *infection as the clinical symptoms are similar (hemoptysis, weight loss, fever, and night sweats). Furthermore, a misdiagnosis could be made due to a lack of clinical suspicion, and, most crucially, the infection is chronic enough to be confused with pulmonary tuberculosis [3].

## Case presentation

**Figure 1:**
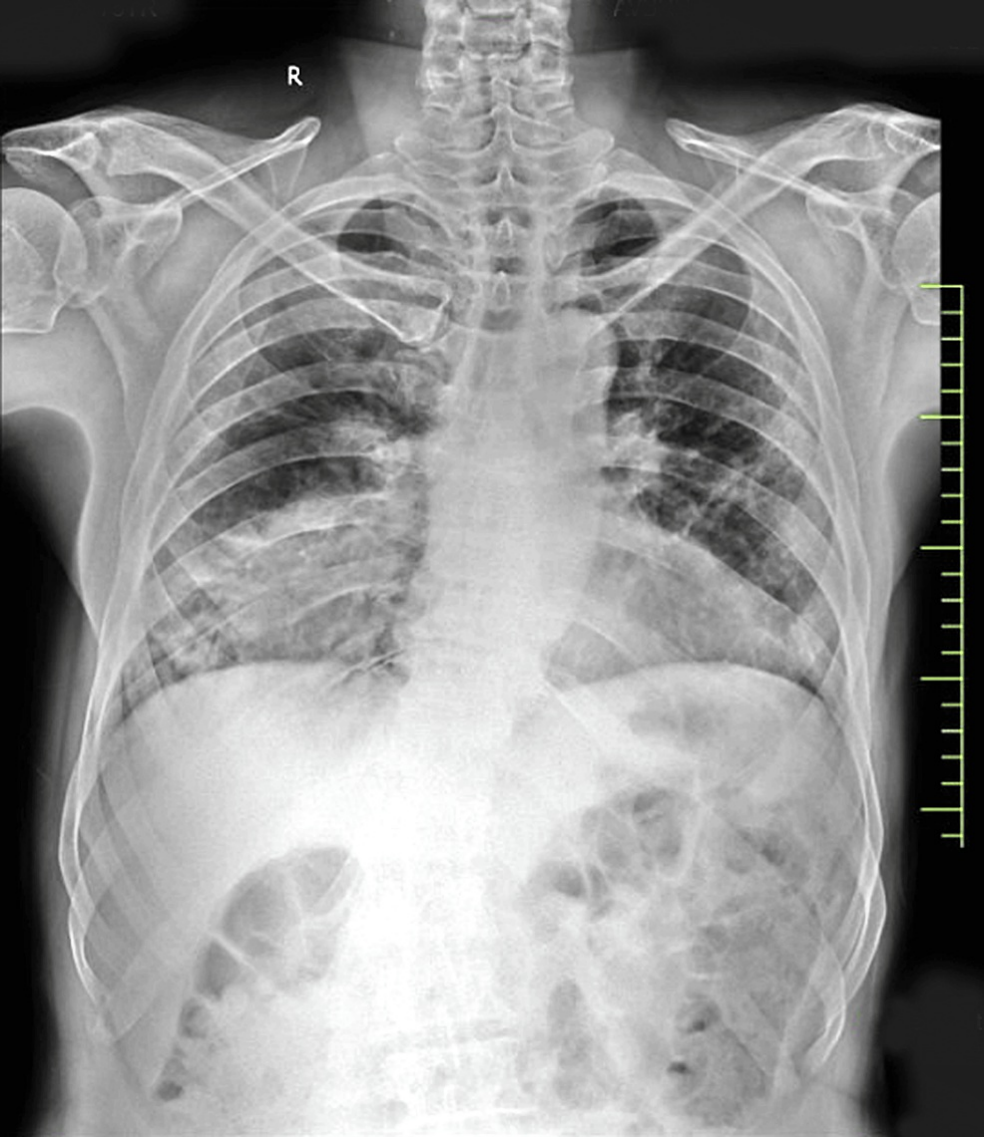
Chest radiograph of the patient on presentation. Homogenous rounded consolidation in the right lower zone of the lung.

A 75-year-old male patient from a rural area with no underlying comorbidities was diagnosed with pulmonary tuberculosis two years ago, for which he had taken anti-tubercular therapy (ATT) for two months only and then stopped the medication. He presented to us with the chief complaints of cough with mucoid expectoration for four months. Coughing was associated with chest pain in the right inframammary region. The cough was also associated with mild hemoptysis and was not relieved by a cough suppressant. The patient also complained of breathlessness for four months, progressing from grade 1 to grade 3 mMRC, which was aggravated by exertion and relieved by rest. The patient complained of intermittent low-grade fever for four months. He gave a history of exposure to biomass of plant origin. The patient also complained of weight loss with no change in appetite. The patient had a history of paroxysmal nocturnal dyspnea, which was relieved by sitting in a flexed posture. The patient was sleep-deprived due to cough and breathlessness. On examination, blood pressure was 108/72 mmHg, pulse rate was 69 beats/minute, respiratory rate was 18 breaths/minute, and oxygen saturation was 96% in room air. Upon physical examination, respiratory sounds in the right lower lobe were decreased. A chest X-ray in an erect position in the posteroanterior view revealed homogenous rounded consolidation in the right lower zone of the lung (Figure 1).

**Figure 2:**
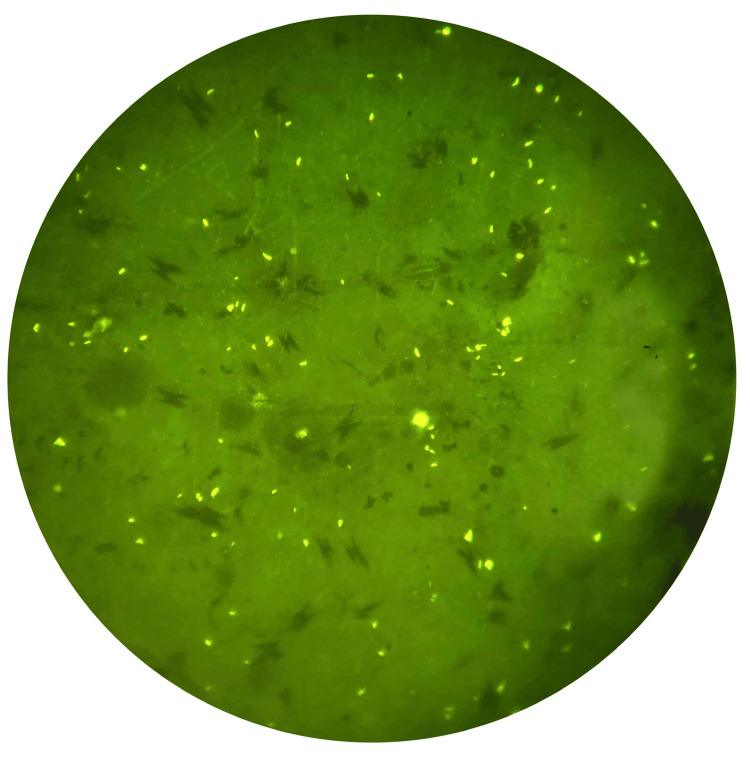
Mycobacterium bacilli stained with fluorescent auramine-rhodamine stain.

Subsequent sputum examination showed positive results for auramine-rhodamine fluorescent stain for *Mycobacterium tuberculosis* (Figure 2). Rifampicin resistance was not detected. Blood investigations showed hemoglobin of 9.3 g/dL (reference range = 12-16 g/dL). Serum IgE levels were 1,127 ng/mL. Serum IgG specific for *Aspergillus fumigatus* was positive. The remaining pertinent laboratory results are shown in Table 1 and Table 2.

**Table 1:** Complete blood count investigations with cell counter with peripheral smear. MCHC = mean corpuscular hemoglobin concentration; MCV = mean corpuscular volume; MCH = mean cell hemoglobin; RDW = red cell distribution width

Lab variables	Results	Reference range
Hemoglobin (g/dL)	9.3	12–16
MCHC (g/dL)	31.7	31–35
MCV (fL)	75.9	76–100
MCH (pg/cell)	24.1	25–32
Total RBC count (million cells/mm^3^)	3.88	0–1.070
Total WBC count	7,900	3,500–9,000
Total platelet count (× 10^5^)	3.99	1.5–4.5
Hematocrit (%)	29.5%	42–53
Granulocytes ((× 10^9^)	5.5	1.5–8.5
Lymphocytes (%)	40	25–45
RDW (%)	18.1	11.5–14.5
Monocytes (%)	04	3–7
Eosinophils (%)	01	1–3
Basophils (%)	00	0–0.75

**Table 2:** Liver function test, kidney function test, and other relevant tests. SGPT = serum glutamic-pyruvic transaminase; SGOT = serum glutamic-oxaloacetic transaminase; A/G ratio = albumin to globulin ratio; HCV = hepatitis C virus; HbsAg = hepatitis B surface antigen; HIV = human immunodeficiency virus

Lab variables	Results	Reference range
Liver function test		
Alkaline phosphatase (U/L)	100	53–128
SGPT (U/L)	11	7–56
SGOT (U/L)	20	8–33
Total protein (g/dL)	8.3	6.0–8.3
Albumin (g/dL)	3.2	3.4–5.4
Globulin (g/dL)	5.1	2.0–3.5
A/G ratio serum	0.62	1.3–2
Total bilirubin (mg/dL)	0.5	0.1–1.0
Conjugated bilirubin (mg/dL)	0.2	0–0.3
Unconjugated bilirubin (mg/dL)	0.3	0.2–0.8
Kidney function test		
Urea (mg/dL)	28	6–24
Creatinine (mg/dL)	1.1	0.5–1.5
Sodium (Na^+^) serum (mEq/L)	139	135–145
Potassium (K^+^) serum (mEq/L)	4.5	3.5–5.0
Other relevant tests		
Calcium serum (mg/dL)	8.4	8.4–10.2
Magnesium (mg/dL)	1.7	1.5–2.0
Phosphorus serum (mg/dL)	5.0	2.8–4.5
Anti-HCV (rapid)	Non-reactive	-
HbsAg (rapid)	Non-reactive	-
HIV (rapid)	Non-reactive	-

**Figure 3:**
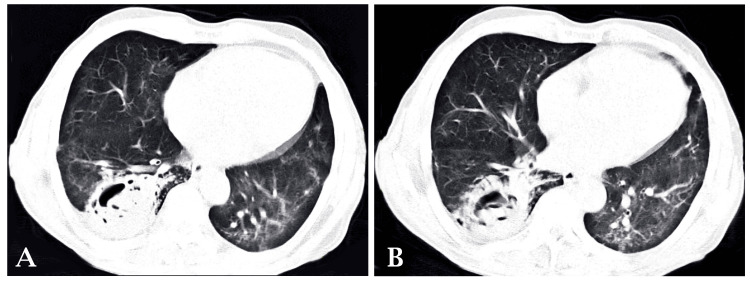
Computed Tomography Scan of the Chest of the Patient at Presentation (A) evidence of crescent-shaped lucency around the soft tissue and (B) Posterobasal segment of the right lower lobe with adjacent areas of ground glass opacities and soft tissue opacification within the cavity.

After an X-ray and laboratory examination, a further radiological examination with high-resolution computed tomography (HRCT) was done (Figure 3). HRCT of the lungs revealed a cavitary lesion measuring approximately 4 × 6 × 5.1 cm in the posterobasal segment of the right lower lobe with adjacent areas of ground-glass opacities and soft tissue opacification within the cavity, evidence of crescent-shaped lucency around the soft tissue strongly supporting mycetoma. Multiple areas of centrilobular nodules arranged in linear branching patterns showed bud appearance in the right lower lobe due to active infective etiology.

Initially, the patient was started on broad-spectrum antibiotics (ceftriaxone), antipyretics, and cough suppressants. Cough suppressants showed minimal effectiveness. For mild hemoptysis, the patient was prescribed etamsylate, tranexamic acid, and vitamin K, which immediately decreased the episodes of hemoptysis. After confirming the diagnosis of tuberculosis, the patient started ATT, which included a combination of isoniazid, rifampicin, pyrazinamide, and ethambutol. After the patient was confirmed to have mycetoma (aspergilloma), he was started on oral Itraconazole. The patient showed good symptomatic improvement and was discharged and asked to follow up after one month.

## Discussion

Pulmonary tuberculosis can cause remodeling of the lung parenchyma through various processes, including pulmonary cavitation, pulmonary fibrosis, bronchiectasis, airflow obstruction, restrictive ventilatory defects, and impaired gas exchange. Patients with pulmonary tuberculosis, especially those with secondary tuberculosis, develop cavitary lesions susceptible to *Aspergillus *infection [4,5]. Depending on the changes in the lung, parenchyma aspergillosis can manifest as saprophytic aspergilloma (mycetoma), allergic bronchopulmonary aspergillosis, and invasive aspergillosis [6]. This type of change leads to dead space in the lung parenchyma, devoid of the blood supply, providing a suitable environment for fungal infections such as aspergillosis in the form of mycetoma can also be called aspergilloma or fungal ball [7]. Chest CT shows a cavitary lesion in the posterobasal segment of the right lower lobe with adjacent areas of ground-glass opacities and soft tissue opacification within the cavity [8]. Multiple areas of centrilobular nodules arranged in linear branching patterns show a tree-in-bud appearance in the right lower lobe due to active *Aspergillus *infection [9]. The patient started taking broad-spectrum antibiotics, antipyretics, and cough suppressants for symptomatic relief. After confirmation of pulmonary tuberculosis, the patient started taking ATT while continuing the earlier prescription for symptomatic relief. After confirmation of mycetoma, the patient was started on oral itraconazole. Treatment proved to be effective and symptomatic relief was seen in the patient. Taking into account the symptomatic improvement, the patient was discharged and asked to follow up after one month.

## Conclusions

Notably, the simultaneous isolation of *Aspergillus *species and *Mycobacterium tuberculosis* from respiratory samples is challenging and needs careful consideration to prevent adverse outcomes. Along with the treatment for symptomatic relief of tuberculosis, the patient was started with an ATT regimen. As for mycetoma, pharmacotherapy with itraconazole has a minimal role because the lungs are already damaged. Moreover, mycetoma cannot be removed as the infected part does not have enough blood supply for the effective action of pharmacotherapy provided by drugs. Taking into account these factors, mycetoma must be removed surgically by right lower lobectomy for complete eradication of the infected part. Clinical regression and symptom improvement were observed in the patient. Accurate diagnosis and treatment with a multidisciplinary team within limited time and resources can be the difference between morbidity and mortality.
